# Stereotactic body radiation therapy (SBRT) for definitive treatment and as a bridge to liver transplantation in early stage inoperable Hepatocellular carcinoma

**DOI:** 10.1186/s13014-017-0899-4

**Published:** 2017-10-19

**Authors:** Assaf Moore, Michal Cohen-Naftaly, Anna Tobar, Yulia Kundel, Ofer Benjaminov, Marius Braun, Assaf Issachar, Eytan Mor, Michal Sarfaty, Dimitri Bragilovski, Ran Ben Hur, Noa Gordon, Salomon M. Stemmer, Aaron M. Allen

**Affiliations:** 1 Institute of Oncology, Davidoff Center, Rabin Medical Center, Petah Tikva, Israel; 2 Sackler Faculty of Medicine, Tel Aviv University, Tel Aviv, Israel; 3 Liver Institute, Rabin Medical Center, Petah Tikva, Israel; 4 Department of Imaging, Rabin Medical Center, Petah Tikva, Israel; 5 Department of Organ Transplantation, Rabin Medical Center, Petah Tikva, Israel; 6 Department of Pathology, Rabin Medical Center, Petah Tikva, Israel

**Keywords:** Hepatocellular carcinoma (HCC), Stereotactic body radiotherapy (SBRT), Liver transplantation

## Abstract

**Background and Purpose:**

Stereotactic body radiotherapy (SBRT) is an emerging modality for definitive treatment of Hepatocellular carcinoma (HCC).

**Materials and Methods:**

This retrospective study included all early stage HCC patients who were not candidates for primary resection and/or local therapy, treated with SBRT between 11/2011 and 1/2016.

**Results:**

Twenty-three patients were included. The median age was 62 years; 70% males; 30% females; 70% viral hepatitis carriers; 100% cirrhotic; 13 Child Pugh [CP]-A and 10 [CP]-B. The median tumor volume was 12.7cm^3^ (range, 2.2–53.6 cm^3^). Treatment was well tolerated. With the exception of one patient who developed RILD, no other patient had significant changes in 12 weeks of laboratory follow-up. SBRT was a bridge to transplantation in 16 patients and 11 were transplanted.. No surgical difficulties or complications were reported following SBRT, and none of the transplanted patients had local progression before transplantation. The median prescribed dose to the tumor was 54Gy (range, 30-54Gy), the median dose to the uninvolved liver was 6.0Gy(range, 1.6–12.6Gy). With a median follow-up time of 12 months, the median overall-survival for the 11 transplanted patients was not reached (range, 2.0–53.7+ months) and was 23 months for the 12 non-transplanted patients. The median progression-free survival for the transplanted patients was not reached (54+ months) and was 14.0 months for the non-transplanted patients. There was no SBRT-related mortality. Liver explant post SBRT revealed pathological complete response in 3(27.3%), pathological partial response in 6(54.5%), and pathological stable disease in 2(18.2%) tumors.

**Conclusions:**

SBRT is safe and effective and can be used as a bridge to transplantation without comprising the surgical procedure.

## Background

Hepatocellular carcinoma (HCC) is the sixth most common malignancy, and the third most common cause of cancer mortality worldwide [1]. HCC incidence has been rising steadily due to increasing numbers of hepatitis C virus (HCV) carriers [1]. Unique staging methods have been developed, such as the Barcelona-Clinic Liver Cancer staging classification (BCLC), which relates to the Child Pugh (CP) score of liver function, as well as to the patient’s performance status as evaluated by the Eastern Cooperative Oncology Group (ECOG) score [2, 3]. The therapeutic recommendations are often based on the BCLC staging system, which reflect prognosis and survival. BCLC stage A patients are candidates for surgical procedures, such as resection or liver transplantation [3, 4]. As not all transplant candidates can be transplanted in a timely manner due to shortage in organs, bridging treatments may be required. Invasive locoregional therapies such as transatrerial chemoembolization (TACE) and radiofrequency ablation (RFA) have been studied for this indication [5–9].

These locoregional therapies are the treatment of choice for unresectable lesions or in patients with poor performance status but with sufficient hepatic reserve [4, 10, 11]. TACE is the first-line palliative treatment for unresectable or multi-focal HCC in the absence of metastases, and is recommended for BCLC stage B patients [3, 4, 10, 11].

Previously, external beam radiation therapy (EBRT) was limited by the risk of radiation-induced liver disease (RILD). With advances in imaging and radiotherapy (RT) technologies, conformal liver irradiation has become a feasible and safe technique for treating focal HCC, with RILD rates of <5% [12]. Several studies have established the efficacy and safety of conformal liver irradiation for treating HCC [13–15]. Daily doses ranged from 1.5–5 Gy to total doses of 40–90 Gy, with higher doses associated with improved outcomes [13–15]. Specific dose volume tolerances have been defined for conventional fractionation.

Stereotactic body radiotherapy (SBRT) is a technique that delivers higher doses of radiation and has been explored for the treatment of HCC. However, most of the data arise from patients who have Child-Pugh A (CP-A) liver disease [16–18]. SBRT in patients who are classified as Child-Pugh B (CP-B) liver disease but requires some modifications and strict adherence to dose constraints [19]. SBRT has also been studied as a bridge to liver transplantation with promising results, but data is scarce [20, 21]. Our study focuses on the use of SBRT as a bridge for transplant as well as on dose escalation in CP-B patients.

## Methods

### Patients

The study included all consecutive HCC patients who were not candidates for primary resection and/or local therapy and who were treated with intensity-modulated radiation therapy (IMRT)/image-guided radiation therapy (IGRT)-based SBRT at the Institute of Oncology between November 2011 and January 2016. All cases were discussed in a multidisciplinary tumor board including hepatologists, hepatobiliary/transplant surgeons, medical oncologists, radiation oncologists and radiologists as well as invasive radiologists.

### Data collection and outcomes

This retrospective study was approved by the medical center institutional Helsinki review board. No informed consent was required. Data were collected from medical records and included demographics, viral status, history of cirrhosis and underlying liver disease, tumor location and size, performance status, response to treatment, survival, and cause of death. Liver enzyme, albumin, creatinine and total bilirubin levels, as well as coagulation studies (international normalized ratio [INR]) were recorded before and every 2 weeks after completion of radiotherapy up to 12 weeks. Treatment outcome was assessed by liver magnetic resonance imaging (MRI) or triphasic computed tomography (CT) scan. Tumor response in patients who underwent liver transplantation was assessed by explant pathology. Progression-free survival (PFS) was defined as the time between SBRT and the first imaging study that indicated disease progression – local or distant. Local progression was defined as an increase of 20% or more of the primary tumor volume. Local control was defined as the lack of local progression. Overall survival (OS) was calculated from the time of SBRT. Treatment-related death was defined as death within 30 days of treatment. The surgical report and post-op charts were reviewed for remarks on surgical difficulty and vascular complications.

### Treatment planning

Patients were immobilized for simulation with a customized vacuum cushion and abdominal compression was applied. Patients were simulated using a multiphase 4-dimentional CT simulation to monitor breathing-related liver motion. Images were reconstructed on the Advantage Workstation (GE Healthcare, Chicago, IL). When anatomically feasible, fiducial markers were placed. The internal target volumes (ITVs) were created using all 10 phases to account for maximal tumor excursion.

The gross tumor volume (GTV) was defined as the contrast enhancing tumor volume on a triphasic CT or MRI scan. The planning treatment volume (PTV) was defined as a 3-mm margin around the GTV, after expansion for ITV was made. The PTV was reduced in case of proximity to vital normal tissue. Patients were treated with IMRT using dynamic sliding window multileaf collimator (MLC) or volumetric modulated arc therapy (VMAT). Specification of the dose-volume histogram (DVH) constraints is available in Table 1.

**Table 1:** DVH constraints for organs at risk

Organ	Constraints
Uninvolved Liver	V5 < 50%, V7 < 30%, V15 < 700 cm^3^ For Child Pugh B – mean liver dose <10 Gy
Rt. Kidney	V15 < 35%
Small Bowel	max dose <30Gy
Spinal Cord	max dose <18Gy

Dose calculations were performed using the Eclipse™ treatment planning system (Varian, Palo Alto, CA), AAA algorithm version 8. Treatment was prescribed to the 95% isodose line with PTV tolerance of ±5%. Quality assurance verification plans were performed with the ArcCHECK™ dosimeter (Sun Nuclear Corporation, Melbourne, FL).

Before each treatment, cone beam CT (CBCT) was used to position the patient appropriately. Doses of 30, 48 and 54 Gy in 5, 4 and 3 fractions respectively were delivered every other day. Dose of radiotherapy was recommended to be 54 Gy for patients who were CP-A and 30 Gy in 5 fractions for CP-B. Some patients also received 48 Gy in 4 fractions when normal tissue constraints could not be met.

### Post SBRT evaluation

The treated tumors were assessed by MRI or triphasic CT eight weeks from completion of SBRT. Following imaging studies were scheduled at the treating physician’s discretion. For the patients in whom alpha-fetoprotein level was elevated before SBRT, repeat tests were done monthly. In cases where the tumor marker indicated progression, an imaging study was performed.

### Pathological workup

The liver explants were cut in slides in an attempt to reproduce the sections obtained in imaging and to receive correlation with the site of the nodules. Sections from each major tumor nodule and representative samples of small nodules were taken. Satellites nodules, multifocal HCC and intrahepatic metastasis were not distinguished and were considered multiple tumors. Tumor grade and extension were evaluated, including extension into the hilar vessels, inferior vena cava, the hepatic capsule, and margins.

The liver sampling consisted of: (1) Hepatic hilus including hepatic artery, portal vein, and bile duct; (2) Inferior vena cava; (3) Sections from the nodules with the detail of the location; and (4) Section of the cirrhotic liver from the left and right lobes. Conventional hematoxylin-and-eosin stain was performed, as well as special stains, including: Periodic acid–Schiff, Periodic acid–Schiff–diastase, Masson’s trichrome, Reticulum, Ferrum, Orcein, Keratin 19 and 7.

### Statistical analysis

Data were analyzed using the Statistical Package for the Social Sciences 22.0 (SPSS) at a significance level of 0.05. Survival was estimated using the Kaplan-Meier method and Cox’s regression analysis.

## Results

### Patient characteristics

The study included 23 patients. Median age at RT was 62.2 (range, 43.0–83.9) years, 69.6% were male and 30.4% female, 70% were viral hepatitis carriers, all had liver cirrhosis. All patients were either CP-A (56.5%) or CP-B (43.5%) and ECOG performance status ≤2. All patients had early stage disease (BCLC stage A). CP-B score was determined based on biochemical findings, no patient had encephalopathy or ascites before being treated with SBRT. Six of the CP-B patients were CP-B score 7 (CP-B7), and 4 were CP-B score 8 (CP-B8). Two patients were previously treated with invasive locoregional methods. In 21 patients, SBRT was the first treatment modality (Table 2).

**Table 2:** Patient characteristics

Characteristics	All Patients (*n* = 23)
Median age at diagnosis, year (range)	62.2 (43.0–83.9)
Male, n (%)	16 (69.6%)
Female, n (%)	7 (30.4%)
Liver disease, n (%)	
NASH Cirrhosis	5/23 (21.7%)
HBV Cirrhosis	4/23 (17.4%)
HCV Cirrhosis	12/23 (52.2%)
Cirrhosis NOS	2/23 (8.7%)
Child-Pugh, n (%)	
A	13/23 (56.5%)
B	10/23 (43.5%)
Stage at diagnosis, n (%)	
BCLC A	23/23 (100%)
Transplantation candidates, n (%)	
Yes	16/23 (69.5%)
No	7/23 (30.5%)

NASH *Nonalcoholic steatohepatitis*, HBV *hepatitis B virus*, HCV *hepatitis C virus*, NOS *not otherwise specified*, BCLC *Barcelona-Clinic Liver Cancer Staging Classification*

### Treatment parameters

The median number of lesions was 1 (range, 1–4), the median tumor diameter was 2.5 (range 1.0–4.8) cm, and the median tumor volume was 12.7 (range 2.2–53.6) cm^3^. The median prescribed dose to the tumor was 54 (range, 30–54) Gy. The median of the mean dose to the uninvolved liver (mean liver dose, MLD) was 6.0 (range, 1.6–12.6) Gy. The median V5/V7/V15 to the uninvolved liver was 33.4% (range, 6.2–48.4%), 27.2% (range, 5.7–44.9%), and 14.6% (range, 2.9–27.0%) respectively. The median mean dose to the right kidney and to 1 cm^3^ bowel was 0.4 (range, 0.05–8.29) Gy and 7.8 (range, 0.1–17) Gy, respectively.

### Survival

**Fig. 1:**
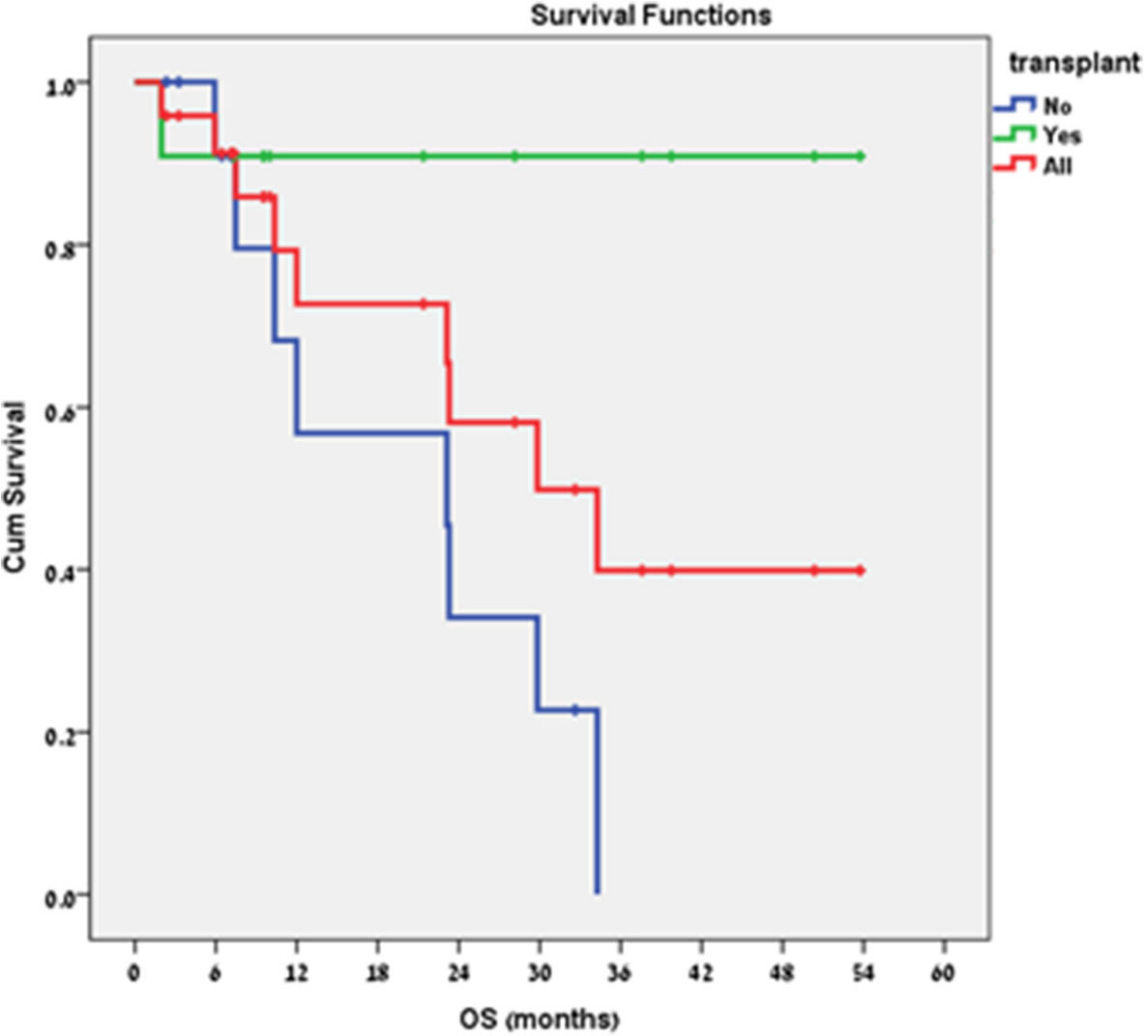
Overall Survival. Median OS: entire cohort - 34.2 (range, 2.0–53.7) months, non-transplanted patients - 23.3 (range, 2.3–34.3) months, transplanted patients - not reached

After a median follow-up time following RT of 12 (range, 2.0–48.7) months, the median OS for the entire cohort was 34.2 (range, 2.0–53.7) months. The median OS for patients who were not transplanted was 23.3 (range, 2.3–34.3) months and was not reached for the transplanted patients (Fig. 1). There was no 30 day radiation-related mortality.

### Progression free survival

**Fig. 2:**
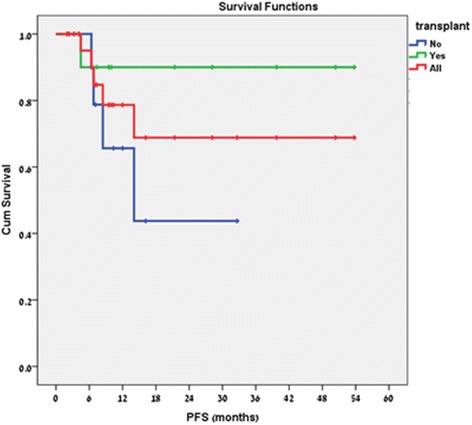
Progression Free Survival. Median PFS: non-transplanted patients - was 14.0 (range, 2.3–32.6) months, transplanted patients - not reached

The median PFS for the entire cohort and for the transplanted patients was not reached (as there were too few events of disease progression), and was over 54 months (Fig. 2). The median PFS for patients who were not transplanted was 14.0 (range, 2.3–32.6) months (Fig. 2).

### Toxicity

Two patients had grade 1upper gastrointestinal toxicity with abdominal discomfort and grade 1 weakness and fatigue. One patient (CP-B8, treated to 30 Gy) developed RILD two weeks after SBRT that manifested in rising serum total bilirubin levels and INR, as well as new ascites. This patient’s hepatic decompensation was determined to be RILD be a multidisciplinary team. The V15 and mean dose to the uninvolved liver were 9.16 Gy and 5.08 Gy respectively. This patient subsequently underwent urgent and successful transplantation. No other patient had significant changes in 12 weeks of laboratory follow-up.

### Transplanted patients

**Fig. 3:**
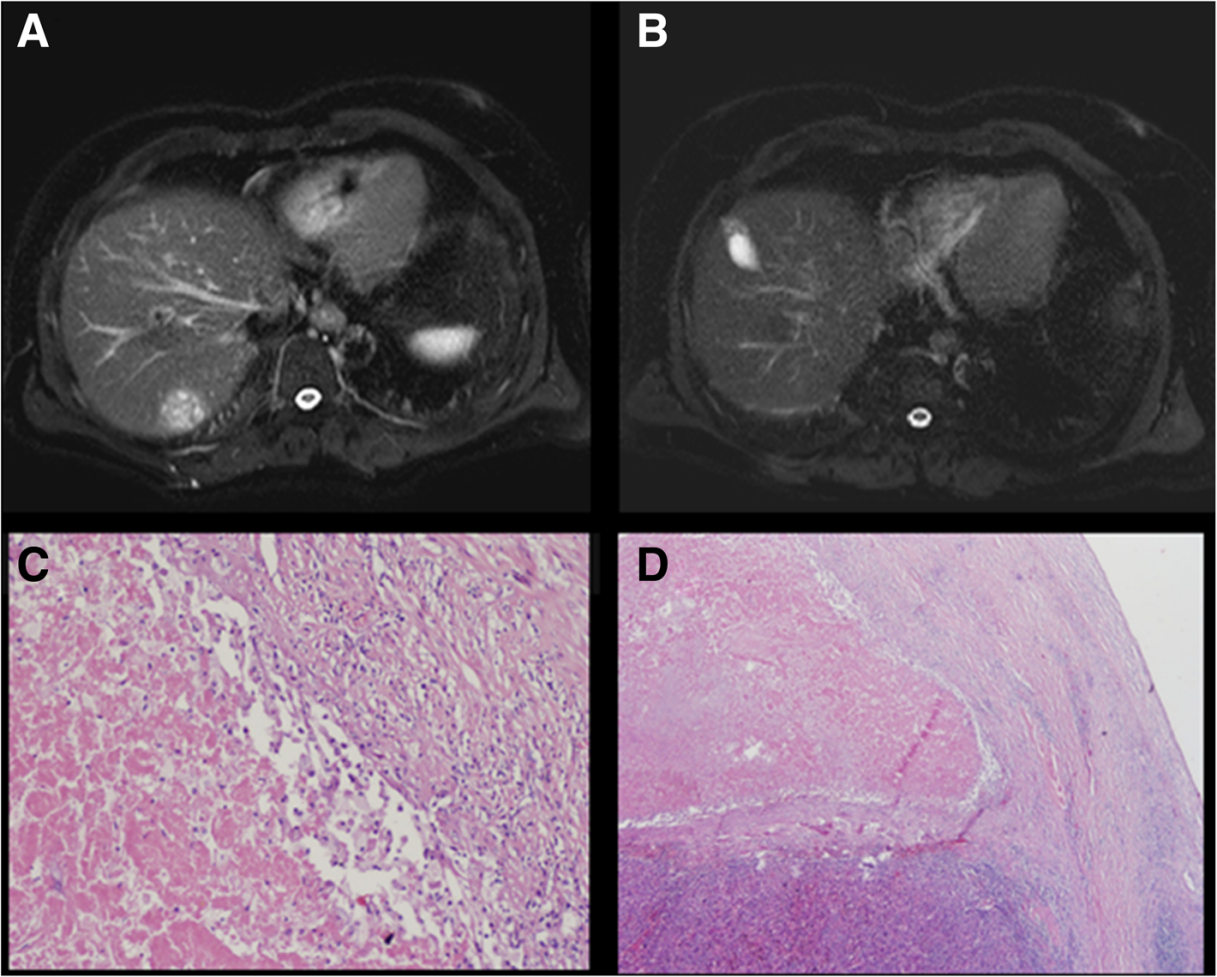
**a** A pre-treatment MRI scan of an HCC nodule in May 2013 (**b**) An MRI scan in May 2016, demonstrating a durable tumor response to SBRT (**c** + **d**) Liver explant pathology from an HCC nodule treated with SBRT, demonstrating areas for coagulative necrosis and areas of loss of tumor morphology

Eleven of 16 eligible patients (68.7%) underwent orthotopic liver transplantation. The surgical teams reported no significant surgical difficulties. All tumors conformed to the Milan Criteria. The median time to transplantation was 4.8 (range, 0.16–8.5) months. Of these 11 patients, 3 (27.3%) achieved pathological complete response (pCR), 6 (54.5%) achieved pathological partial response (pPR), and 2 (18.2%) achieved pathological stable disease (pSD) (Table 3). Local control was achieved in all patients. A patient who was transplanted 5 weeks after SBRT died of sepsis 4 weeks after transplantation. This patient did not experience any surgical/radiation-related complications and died from antibiotic-resistant sepsis while in the intensive care unit (Fig. 3 and Table 4).

**Table 3:** Treatment parameters

number of lesion, median (range)	1 (1–4)
tumor diameter, median cm (range)	2.5 cm (1.0–4.8 cm)
tumor volume, median cm^3^ (range)	12.7 cm^3^ (2.2–53.6 cm^3^)
prescribed dose, median Gy (range Gy)	54 Gy (30–54 Gy)
mean liver dose, median Gy (range Gy)	6.0 Gy (1.6–12.6 Gy)
V5 to uninvolved liver, median % (range %)	33.4% (6.2–48.4%)
V7 to uninvolved liver, median % (range %)	27.2% (5.7–44.9%)
V15 to uninvolved liver, median % (range %)	14.6% (2.9–27.0%)

**Table 4:** Child Pugh score, Tumor Location, radiation dose and fractionation and explant pathology in the 11 transplanted patients

Patient #	Child pugh score	Involved Liver segment/s	Prescribed dose, fractionation	Pathological response
1	A	7	54Gy, 3 fractions	pCR
2	A	4	54Gy, 3 fractions	pCR
3	B	4	48Gy, 4 fractions	pCR
4	A	6/7	54Gy, 3 fractions	pPR
5	A	7/8	54Gy, 3 fractions	pPR
6	B	6	54Gy, 3 fractions	pPR
7	B	2	54Gy, 3 fractions	pPR
8	A	4	48Gy, 4 fractions	pPR
9	B	4	30Gy, 5 fractions	pPR
10	B	6	30Gy, 5 fractions	pSD
11	B	7	30Gy, 5 fractions	pSD

pCR *pathological complete response*, pPR *pathological partial response*, pSD *pathological stable disease*

### Non-transplanted patients

Of the 12 patients who were not transplanted, 7 (58.3%) were not candidates for transplantation (elderly patients or beyond the Milan Criteria) and 5 (41.7%) are awaiting transplantation. All 5 patients awaiting transplantation are without progression with a median time from SBRT completion of 6.7 (range, 2.7–32.9) months. Of the 7 non-transplant candidates, 5 (71.4%) had progression within a median time of 6.8 (range, 4.1–14.1) months. Two patients developed a new lesion in the liver - one was re-irradiated, was progression-free for 9 months after re-irradiation, and then died of hepatic decompensation. Another was treated with TACE. Two patients had systemic progression, and one had local progression that was not treated due to poor PS. The 2 remaining patients died of hepatic decompensation and variceal bleeding (10 and 12 months after RT) without tumor progression.

## Discussion

Patients with early stage HCC (BCLC A) are candidates for potentially curative treatment [3]. Liver transplantation is the best therapeutic option as both the underlying liver disease and tumor can be cured [3]. Invasive ablativemethods have been traditionally used as a curative option for small or inoperable tumors, as well as bridging treatments to prevent disease progression that may render patients ineligible for transplantation [5–8, 10]. These methods, such as TACE and RFA are usually performed in an in-patient setting.

RFA as bridging treatment before transplantation was found to be well tolerated, and produced a CR of 55% (5). However, complete extinction of the tumor is highly dependent on tumor size (5). In one study, bridging TACE before transplantation produced tumor necrosis ≥90% on32–44% of patients treated and three-year recurrence-free survival of 61–87% (8). The most common complication of TACE is postembolization syndrome – which is a self-limited event, but may extend hospital stay significantly (9). The most dreaded complications are liver failure and emboli affecting distant sites (9). The major concern with liver irradiation is RILD, especially in patients who were CP-B.

Advances in RT technique and planning as well as organ motion monitoring have enabled dose escalation while minimizing exposure of uninvolved tissues. SBRT is commonly used for the treatment of primary tumors as well as metastatic lesions in a variety of indications, and data have been accumulating about the use of SBRT in various HCC stages. Some studies have explored SBRT for small inoperable tumors, large tumors, or cases unsuitable for standard invasive locoregional therapies. In a phase II study of 50 inoperable patients previously treated with transarterial chemoembolization (TACE) 1 to 5 times, 38.3% of patients receiving SBRT achieved PR or CR at 6 months. The 2-year control rate, OS and PFS were 94.6%, 68.7% and 33.8%, respectively [16]. In a study of 42 patients with small inoperable HCC, CR was achieved in 33%. The 1 and 3-year-OS were 92.9% and 58.6%, respectively [17]. An additional sequential phase 1 and 2 trials, included 102 patients with HCC unsuitable for locoregional therapy, of whom 61% had multiple lesions, 55% had tumor vascular thrombosis, and 12% had extrahepatic disease. All patients were treated with SBRT with a local control rate at 1-year of 87% and median OS of 17.0 months [18].

All patients in our current study were considered ineligible for primary resection and invasive methods due to tumor size, location, and liver function. All patients included in our study had liver cirrhosis, and 43% were CP-B. Dose constraints were strictly maintained with specific parameters for CP-B patients. All patients who were CP-A and 6 of 10 patients who were CP-B were treated to 48 or 54 Gy. Four of the 10 CP-B patients were treated to a lower dose of 30 Gy. With the exception of 1 case of suspected RILD in a patient who was CP-B treated to 30 Gy, no significant toxicities were observed, which may indicate that dose escalation for CP-B can be accomplished relatively safely.

Conformal RT has also been studied as a bridge to liver transplantation, however data are lacking. In a series of 10 transplantation candidates who failed or were not suitable for standard local therapies and were treated with 5–6 fractions to a median dose of 33 (range, 8.5–54) Gy, explant pathology was available for the 5 patients who were eventually transplanted. The analysis demonstrated 0%, 40%, 60%, 90% and 90% tumor necrosis [22]. In another series of 10 transplantation candidates treated with SBRT, 5-year survival and PFS were 100%. The CR rate was 27% at a median dose of 51 Gy in 3 fractions [20]. Facciuto et al. reported on 27 transplantation candidates who were treated with SBRT of whom 17 eventually were transplanted. In this series, 22 lesions were pathologically evaluated with a 37% response rate (RR): 14% pCR, 23% pPR, and 63% pSD. One patient suffered liver decompensation [20].

Many questions still remain regarding the safety and utility of SBRT for HCC patients, as well as using concomitant biological therapy to improve its efficacy. A recent phase 1 study of 16 CP-A, ECOG performance status 0–1 patients tried to determine the maximal tolerated dose of sorafenib delivered before, during, and after SBRT for HCC. Significant toxicity was observed when the effective irradiated liver volume was high (30–60%) [23]. RTOG 1112 is a randomized phase 3 study comparing sorafenib to SBRT followed by sorafenib in patients with BCLC B or C, unsuitable for surgical or invasive procedures. The results of this pivotal trial are eagerly awaited. The use of particle beams was assessed in a recent phase 2 study, where 44 patients with unresectable HCC, treated with high dose hypofractionated proton beam therapy (median dose, 58 Gy). The local control rate and OS at 2 years was 94.8% and 63.2%, respectively [24]. No randomized trials have compared particle beams with photons and therefore there is no consensus on preference of ion therapy over traditional photons.

In our study, SBRT was effective for local control in non-transplanted patients, similar to results seen in previous studies [16–18, 25, 26]. All were treated as out-patients, and none were admitted for complications. Of the 5 patients who progressed in our cohort, 1 patient who was CP-B developed a second HCC, was re-irradiated without radiation-associated toxicity and remained without progression and had no treatment related complications. To our knowledge, there are no data regarding re-irradiation with SBRT for patients who are CP-B.

Sixteen patients were considered candidates for transplantation. All were treated as out-patients. Eleven patients were eventually transplanted. Within a median time from SBRT to transplantation of 4.8 months none had progression. There were no radiation-related surgical complications. The dose to the tumor, and response rate as seen in liver explants were similar to those reported in a previous study [20]. We suspect a dose escalation response in our cohort. Of the patients treated with 54 Gy, 2 had pCR and 4 had pPR; of the patients treated with 48 Gy, 1 had pCR and 1 had pPR; and of the patients treated with 30 Gy, 1 had pPR and 2 had pSD. It seems that lower doses could suffice as a bridge for transplant but not as a definitive treatment.

The main limitations of our study include its small size and dose heterogeneity. In addition, the potential for selection bias is also a key limitation of our study. However, as there are few reports on the pathologic evaluation of post-SBRT liver explants and no randomized data exists, our study contributes to the expansion of knowledge regarding this noninvasive minimally toxic out-patient procedure.

Overall, the evidence supporting the use of SBRT in HCC patients, including the present study is encouraging. A formal phase 2 dose escalation study in patients who are CP-B is planned both for definitive therapy and as a bridge for transplant.

## Conclusion

SBRT achieved local control without major adverse events and proved to be an effective and safe bridging treatment to liver transplantation. The significant pathological response rate, including 27% pathological complete response is encouraging. There were no unexpected surgical complications in the patients treated with SBRT, therefore, albeit from a small cohort, we suggest that SBRT may be considered as an alternative to traditional invasive local therapies. The optimal dose and fractionation are yet to be established. The role of SBRT in early stage HCC and specifically in patients who are candidates for transplantation needs to be assessed in large randomized controlled trials. The safety and efficacy of liver re-irradiation with SBRT should also be further explored.

## Abbreviations

BCLC: Barcelona-Clinic Liver Cancer staging classification
CP: Child Pugh
CT – CBCT: Cone Beam
CT: Computed Tomography
DVH: Dose-Volume Histogram
EBRT: External Beam Radiation Rherapy
ECOG: Eastern Cooperative Oncology Group
GTV: Gross tumor volume
HCC: Hepatocellular Carcinoma
HCV: Hepatitis C Virus
IGRT: Image-Guided Radiation Therapy
IMRT: Intensity-Modulated Radiation Therapy
INR: International Normalized Ratio
ITVs: Internal target volumes
MLC: Multileaf Collimator
MRI: Magnetic Resonance Imaging
OS: Overall Survival
PFS: Progression-Free Survival
pPR: Pathological Partial Response
pSD: Pathological Stable Disease
PTV: Planning Treatment Volume
RILD: Radiation-Induced Liver Disease
SBRT: Stereotactic Body Radiotherapy
TACE: Transarterial Chemoembolization
VMAT: Volumetric Modulated Arc Therapy

## References

[1] Jemal A, Bray F, Center MM, Ferlay J, Ward E, Forman D (2011). Global cancer statistics. CA Cancer J Cline.

[2] Okuda K, Ohtsuki T, Obata H (1985). Natural history of hepatocellular carcinoma and prognosis in relation to treatment: study of 850 patients. Cancer.

[3] Llovet JM, Brú C, Bruix J (1999). Prognosis of hepatocellular carcinoma: BCLC staging classification. Semin Liver Dis.

[4] Forner A, Llovet JM, Bruix J (2012). Hepatocellular carcinoma. Lancet.

[5] Mazzaferro V, Andreola S (2004). Radiofrequency ablation of small hepatocellular carcinoma in cirrhotic patients awaiting liver transplantation: a prospective study. Ann Surg.

[6] Tsochatzis E, Burroughs AK (2013). Transarterial embolization as neo-adjuvant therapy pretransplantation in patients with hepatocellular carcinoma. Liver Int.

[7] Graziadei IW, Vogel W (2003). Chemoembolization followed by liver transplantation for hepatocellular carcinoma impedes tumor progression while on the waiting list and leads to excellent outcome. Liver Transpl.

[8] Nicolini D, Vivarelli M (2013). Doxorubicin-eluting bead vs conventional transcatheter arterial chemoembolization for hepatocellular carcinoma before liver transplantation. World J Gastroenterol.

[9] NCCN Guidelines, www.nccn.org

[10] Vogl TJ, Naguib NN, Zangos S (2009). Review on transarterial chemoembolization in hepatocellular carcinoma: Palliative, combined, neoadjuvant, bridging, and symptomatic indications. Eur J Radiol.

[11] Takayasu K, Arii S, Ichida T (2006). Prospective cohort study of transarterial chemoembolization for unresectable hepatocellular carcinoma in 8,510 patients. Gastroenterology.

[12] Feng M, Ben-Josef E (2011). Radiation therapy for hepatocellular carcinoma. Semin Radiat Oncol.

[13] McGinn CJ, Ten Haken RK, Ensminger WD (1998). Treatment of intrahepatic cancers with radiation doses based on a normal tissue complication probability model. J Clin Oncol.

[14] Mornex F, Girard N, Beziat C (2006). Feasibility and efficacy of high-dose three-dimensional-conformal radiotherapy in cirrhotic patients with small-size hepatocellular carcinoma non-eligible for curative therapies— mature results of the French phase II RTF-1 trial. Int J Radiat Oncol Biol Phys.

[15] Robertson JM, McGinn CJ, Walker S (1997). A phase I trial of hepatic arterial bromodeoxyuridine and conformal radiation therapy for patients with primary hepatobiliary cancers or colorectal liver metastases. Int J Radiat Oncol Biol Phys.

[16] Kang JK1, Kim MS, Cho CK, Yang KM, Yoo HJ, Kim JH, Bae SH, Jung DH, Kim KB, Lee DH, Han CJ, Kim J, Park SC, Kim YH (2012). Stereotactic body radiation therapy for inoperable hepatocellular carcinoma as a local salvage treatment after incomplete transarterial chemoembolization. Cancer.

[17] Kwon JH, Bae SH, Kim JY (2010). Long-term effect of stereotactic body radiation therapy for primary hepatocellular carcinoma ineligible for local ablation therapy or surgical resection: stereotactic radiotherapy for liver cancer. BMC Cancer.

[18] Bujold A1, Massey CA, Kim JJ, Brierley J, Cho C, Wong RK, Dinniwell RE, Kassam Z, Ringash J, Cummings B, Sykes J, Sherman M, Knox JJ, Dawson LA (2013). Sequential phase I and II trials of stereotactic body radiotherapy for locally advanced hepatocellular carcinoma. J Clin Oncol.

[19] Cárdenes HR, Johnstone PA (2010). Feasibility trial of stereotactic body radiation therapy for primary hepatocellular carcinoma. Clin Transl Oncol.

[20] O'Connor JK, Goldstein RM (2012). Long-term outcomes of stereotactic body radiation therapy in the treatment of hepatocellular cancer as a bridge to transplantation. Liver Transpl.

[21] Facciuto ME, Wolf DC (2012). Stereotactic body radiation therapy in hepatocellular carcinoma and cirrhosis: evaluation of radiological and pathological response. J Surg Oncol.

[22] Sandroussi C, Dawson LA, Grant DR (2010). Radiotherapy as a bridge to liver transplantation for hepatocellular carcinoma. Transpl Int.

[23] Brade AM, Ng S, Dawson LA (2016). Phase 1 trial of Sorafenib and stereotactic body radiation therapy for Hepatocellular carcinoma. Int J Radiat Oncol Biol Phys.

[24] Theodore S. H, Jennifer Y. W, Andrew X Z (2016). Multi-institutional phase II study of high-dose Hypofractionated proton beam therapy in patients with localized, Unresectable Hepatocellular carcinoma and Intrahepatic Cholangiocarcinoma. JCO Feb.

[25] Louis C, Dewas S, Mirabel X (2010). Stereotactic radiotherapy of hepatocellular carcinoma: preliminary results. Technol Cancer Res Treat.

[26] Seo YS, Kim MS, Yoo SY (2010). Preliminary result of stereotactic body radiotherapy as a local salvage treatment for inoperable hepatocellular carcinoma. J Surg Oncol.

